# Expression of energy metabolism related genes in the gastric tissue of obese individuals with non-alcoholic fatty liver disease

**DOI:** 10.1186/1471-230X-14-72

**Published:** 2014-04-09

**Authors:** Rohini Mehta, Aybike Birerdinc, Lei Wang, Zahra Younoszai, Amir Moazzez, Hazem Elariny, Zachary Goodman, Vikas Chandhoke, Ancha Baranova, Zobair M Younossi

**Affiliations:** 1 Betty and Guy Beatty Obesity and Liver Program, Inova Health System, Falls Church, VA, USA; 2 Center for the Study of Chronic Metabolic Diseases, School of Systems Biology, College of Science, George Mason University, Fairfax, VA, USA; 3 Center for Liver Diseases and Department of Medicine, Inova Fairfax Hospital, Falls Church, VA, USA

## Abstract

**Background:**

Stomach is an integral part of the energy balance regulating circuit. Studies exploring the effects of cross-system changes in the energy homeostasis in stomach tissue are scarce. The proximity of the stomach to liver - the most common secondary target affected by obesity – suggests that these two organs are exposed to each other’s local secretion. Therefore, we aimed at expression profiling of energy metabolism associated genes in the gastric tissue of obese non-alcoholic fatty liver disease (NAFLD) patients.

**Methods:**

A total of 24 patients with histologically-proven NAFLD were included. In the gastric tissue, gene expression profiling of 84 energy metabolism associated genes was carried out.

**Results:**

The accumulation of the fat in the liver parenchyma is accompanied by downregulation of genes encoding for carboxypeptidase E (*CPE*) and Interleukin 1B (*IL1B*) in the gastric mucosa of same patient. In patients with high grade hepatic steatosis, Interleukin 1 beta encoding gene with anorexigenic function, *IL1B* was downregulated. The levels expression of 21 genes, including *ADRA2B*, *CNR1* and *LEP* were significantly altered in the gastric tissue of NAFLD patients with hepatic inflammation. There were also indications of an increase in the opioid signaling within gastric mucosa that may results in a shift to proinflammatory environment within this organ and contribute to systemic inflammation and the pathogenic processes in hepatic parenchyma.

**Conclusions:**

We have shown differential expression of energy metabolism associated genes in the gastric tissue of obese NAFLD patients. Importantly, these gene expression profiles are associated with changes in the hepatic parenchyma as reflected in increased scores for hepatic steatosis, inflammation, fibrosis and NASH. This study suggests the complex interplay of multiple organs in the pathogenesis of obesity-related complications such as NAFLD and provides further evidence supporting an important role for gastric tissue in promoting obesity-related complications.

## Background

Energy balance is regulated by a milieu of hormones, cytokines and neurotransmitters. This homeostatic regulation integrates signals from the central nervous system and various peripheral organs and ensures that despite fluctuations in daily food and energy intake, the variation in day to day weight, in most cases, remains negligent [1]. This system-wide crosstalk suggests that the network balancing appetite and satiety is highly complex and, in part, redundant. Stomach is an integral part of this energy balance regulating circuit and is known to relay satiety signals to the hypothalamus [2].

Interestingly, studies exploring the participation of the stomach in energy homeostasis and the effects of cross-system changes in the energy homeostasis on stomach function are scarce [3-5]. Aside from its obvious role in the digestion and absorption of nutrients, the stomach has endocrine function [3,4]. One of the best examples of the endocrine role of the stomach is seen in its production of the ghrelin, obestatin and leptin, hormones that are known to contribute to many chronic diseases asscociated with obesity [5-7]. Additionally, several recent studies have suggested a role of these molecules in systemic inflammation [8-10]. This indicates the necessity of further studies on the role of gastric tissue in obesity and obesity-related disorders.

Importantly, obesity is associated with changes in gene expression pattern within many types of non-adipose peripheral tissues, including muscle [11], liver [12] and peripheral blood mononuclear cells [13]. Notably, the effect of obesity on the stomach tissue and the role of the stomach in metabolic dysfunction has been largely overlooked. Histological studies of the stomach tissue in obese patients reported a number of visible changes within the mucosa in a majority of samples [14,15]. It is very difficult to say if these changes are sequelae of systemic inflammation or active contributors to weight gain. It is possible that altered secretory patterns associated with gastric inflammation augment the development of obesity-associated conditions. The proximity of the stomach to liver - the most common secondary target affected by obesity – suggests that these two organs are exposed to each other’s local secretion. Thereby, the gene expression responses of these organs in responder to central adiposity can be potentially inter-related.

An important complication of obesity, non-alcoholic fatty liver disease (NAFLD), is estimated to affect ~30% of the US adults [16]. The progressive form of NAFLD or non-alcoholic steatohepatitis (NASH) is characterized by the accumulation of fat in the liver along with ballooning of hepatocytes, lobular inflammation with or without fibrotic changes in hepatic parenchyma. It is important to note that deposition of the fat in the liver is associated with impaired sensitivity to insulin [17,18]. Various adipokines and hormones produced by visceral adipose tissue, gastric tissue and liver tissue can potentially contribute to the development of NAFLD and its progression to NASH [10,12].

In a previous study, we demonstrated an altered pattern of gene expression for cytokine and chemokine encoding genes in the gastric tissue of obese individuals with NAFLD [19]. In this study, we further explore this relationship by gene expression profiling of energy metabolism associated genes in the gastric tissue of obese NAFLD patients.

## Methods

### Samples

Stomach tissue samples were collected after informed consent from morbidly obese NAFLD patients during laparoscopic sleeve gastrectomy. The tissue was snap frozen in liquid nitrogen and stored at −80°C. A liver biopsy was performed at the same time; all biopsies were read by same hepatopathologist. Clinical and laboratory variables from the time of surgery were extracted from medical records (Table 1). Other causes of chronic liver disease were excluded based on negative serology for hepatitis B and C, no reported history of toxic exposure and excessive alcohol consumption (> 10 gram/day in women and >20 gram/day in men) was also considered as an exclusion criteria. No patients were receiving thiazolidinediones (TZDs), proton pump inhibitors or other medications for gastritis as well as those associated with fatty liver. The study was approved by Internal Review Board of Inova Hospital (Federal Assurance FWA00000573).

**Table 1:** Clinical and demographic data of the patient cohorts profiled for expression of obesity-related genes

**Demographic or clinical parameter**	**Mean ± SD, or %**
	**(N = 24)**
BMI (*)	47.96 ± 8.2
AST, (U/L) (*)	24.92 ± 8.37
ALT, (U/L) (*)	30.38 ± 12.88
Total cholesterol, mg/dL (*)	205.08 ± 41.82
HDL, mg/dL (*) Females	50.33 ± 11.85
HDL, mg/dL (*) Males	39.66 ± 41.82
Triglyceride, mg/dL (*)	197 ± 105.39
Age	43.93 ± 10.2
Gender (Females)	79% (N = 19)
Race (caucasian)	67% (N = 16)
Advanced inflammation (score ≥ 3)	54% (N = 13)
NASH	63% (N = 15)
Advanced steatosis	42% (N = 10)
Fibrosis	79% (N = 19)
Steatosis with presence of hepatic inflammation	96% (N = 23)
NASH with presence of hepatic inflammation	62.5% (N = 15)

Values marked by asterisk (*) are given as Mean ± SD. SD: standard deviation; BMI: Body Mass Index; NASH: Non-Alcoholic Steatohepatitis; AST: aspartate aminotransferase; ALT: Alanine transaminase; HDL: High-density lipoprotein.

All liver biopsies were read by same hepatopathologist. Histological features such as portal inflammation, lymphoplasmacytic lobular inflammation, polymorphonuclear lobular inflammation, Kupffer cell hypertrophy, apoptotic bodies, focal parenchymal necrosis, glycogen nuclei, hepatocellular ballooning, and Mallory-Denk bodies were evaluated in the H & E sections. The extent of steatosis was graded based on an estimate of the percentage of tissue occupied by fat vacuoles as follows: 0 = none, 1 = <5%, 2 = 6-33%, 3 = 34-66%, 4 = >66%. NASH was defined as steatosis, lobular inflammation, and ballooning degeneration with or without Mallory Denk bodies, and with or without fibrosis. The extent of various immune cell infiltration such as lymphoplasmacytic cells, polymorphonuclear cells and Kupffer cell hypertrophy was assessed by hematoxylin-eosin (H&E) staining. For each category, scores were assigned based on the following system: 0 = none, 1 = few, 2 = moderate, 3 = many. The extent of hepatic inflammation was determined based on the sum of the above individual scores with a score of ≥ 3 being considered as advanced hepatic inflammation and score of <3 being considered as mild/no hepatic inflammation. Severity of pericellular and portal fibrosis was determined by Masson trichrome staining of the biopsy, respectively. The scoring was as follows: 0 = no fibrosis, 1 = mild fibrosis, 2 = moderate fibrosis, 3 = marked fibrosis. Severity of total hepatic fibrosis was determined based on sum of the individual scores (pericellular and portal fibrosis) with score of ≥ 3 being considered as advanced hepatic fibrosis and score of <3 being considered as mild/no hepatic fibrosis. Patients with hepatic steatosis or NASH were considered to have NAFLD.

### RNA extraction and reverse transcription

Total cellular RNAs were extracted from fundic stomach tissue samples (N = 24) using RNeasy kit (Qiagen, USA) according to manufacturer’s instructions. Concentration and quality of the extracted RNAs were measured using absorbance at 260 nm (A_260_) and 280 nm (A_280_) with GeneQuant1300 spectrophotometer (GE Healthcare, USA). Only mRNA samples with A_260_/A_280_ ratio in range of 1.8 - 2.1 were utilized. Additionally, integrity of each total RNA was evaluated by 1% agarose gel electrophoresis with ethidium bromide. Extracted total RNAs were reverse transcribed to single strand cDNA using RT^2^ first strand kit (Qiagen, USA), per manufacturer’s protocol.

### Quantitative real time PCR analysis

Gene expression profiling experiments were performed on cDNA samples using RT^2^ Profiler PCR Arrays (Qiagen, USA) that include 84 orexigenic, anorexigenic, and energy expenditure related genes and their receptors along with five housekeeping genes, according to the manufacturer's protocol (Additional file 1: Table S1). Quantitative real-time PCR reactions were performed in 96 well PCR format using Bio-Rad CFX96 Real Time System (BioRad Laboratories, USA) with a ramp speed of 1°C/sec. The Real Time PCR mixtures consisted of 1 μL cDNA, 7.5 μL of RT PCR Master mix (Qiagen, USA) in a final volume of 25 μL. The thermal profile of the RT-PCR procedure was repeated for 50 cycles: 1) 95°C for 10 min; 2) 10 s denaturation at 95°C, 15 s annealing at 60°C (amplification data collected at the end of each amplification step); 3) dissociation curve consisting of 10 s incubation at 95°C, 5 s incubation at 65°C, a ramp up to 95°C (Bio-Rad CFX96 Real Time System, USA). Melt curves were used to validate product specificity.

### Analysis of gene expression profiles

The threshold cycle (*C*_*t*_) values were obtained for each gene; only *C*_*t*_ values less than 40 were considered for analysis. *C*_*t*_ values of control wells (genomic DNA control, reverse transcriptase control, positive PCR control) were examined separately and used for determining the quality of the run, according to manufacturer’s recommendations. The average of five housekeeping genes: *B2M*, *HPRT1, RPL13A, GAPD*, and *ACTB,* was used to normalize the *C*_*t*_ values. Relative expression was determined using the delta delta *Ct* method (1). Fold change (2) for each gene was calculated as follows:

(1) $$\Delta \Delta C_{t}=2^{-\Delta C_{t}}$$

(2) $$\mathrm{Fold}\;\mathrm{Change}=\Delta C_{t}^{\mathrm{Expt}}/\Delta C_{t}^{\mathrm{Control}}.$$

### Statistical analysis

The study was designed to detect changes in gene expression in the stomach of patients with advanced forms of NAFLD as compared to those with milder forms. The scorings for each histopathological state were as described above. Following comparisons were performed:

1. Severe disease state as compared to mild/no disease state or

2. Presence of the disease as compared to no disease

The significance of differences in gene expression between the groups was assessed using univariate non-parametric Mann–Whitney tests. Spearman’s coefficients of correlation were used to determine whether two variables co-vary, and to measure the strength of their relationship. The independent effects of significant variables (*P* < 0.05) on advanced inflammation, NASH and steatosis were assessed using multiple stepwise regression analysis, with both the backward and forward stepwise selection procedures.

## Results

A total of 24 patients with histologically-proven NAFLD were included. Clinical and demographic data for patients are summarized in Table 1. In the gastric tissue, gene expression profiling of 84 energy metabolism associated genes (Additional file 1: Table S1) was carried out.

### Gene expression signature associated with advanced hepatic steatosis

When mRNA expression levels were compared in samples of patients with advanced steatosis (Grade ≥ 3) to that with mild or no steatosis (Grade < 3), significant decreases in expression levels of mRNAs encoding *CPE* (−1.88, p < 0.04) and *IL1B* (−2.5, p < 0.05) genes were observed (Table 2).

**Table 2:** List of obesity related genes significantly upregulated in gastric tissues of patients with the following pathological conditions

**Genes**	**Fold regulation**	**P value**
**Advanced steatosis (grade ≥ 3) vs mild/no steatosis (score < 3)**		
*CPE*	−1.8	0.04
*IL1B*	−2.5	0.05
**NASH present vs No NASH**		
*IL1R1*	1.99	0.04
*OPRM1*	2.65	0.02
*SIGMAR1*	3.13	0.03
*THRB*	1.94	0.02
*ZFP91*	3.09	0.01
**Advanced hepatic inflammation (score ≥ 3) vs mild/no hepatic inflammation (score < 3)**		
*ADCYAP1*	5.5	0.04
*ADRA2B*	2.1	0.02
*BDNF*	3.5	0.03
*CNR1*	5.1	0.001
*CNTFR*	3.2	0.02
*GALR1*	2.5	0.04
*GH2*	5.1	0.01
*GRPR*	4.1	0.004
*IAPP*	2.5	0.03
*LEP*	2.3	0.04
*LEPR*	2.3	0.02
*MC3R*	4.1	0.02
*NMB*	2.4	0.04
*NMU*	3.9	0.004
*NMUR1*	6.9	0.03
*PPARGC1A*	4.1	0.006
*PRLHR*	4.2	0.02
*RAMP3*	2.5	0.02
*SIGMAR1*	2.3	0.01
*SSTR2*	3.2	0.04
*UCN*	4.9	0.04
**Fibrosis present vs no fibrosis**		
*NTS*	6.7	0.02
*OPRK1*	5.6	0.01
**Gastritis present vs no gastritis**		
*C3*	−2.1	0.04
*DRD1*	2.6	0.02

Advanced Liver Inflammation (score ≥ 3) (N = 13), Advanced Steatosis (score ≥ 3) (N = 10), NASHǂ (N = 15), Fibrosisǂ (N = 19), Gastritisǂ (N = 11). ǂComparison was performed to groups of patients without the condition listed.

### Gastric gene expression signature associated with NASH

mRNAs encoded by *IL1R1* (1.99, p < 0.04), *OPRM1* (2.65, p < 0.02), *SIGMAR1* (3.13, p <0.03), *THRB* (1.94, p < 0.02) and *ZFP91* (3.09, p < 0.01) genes were upregulated in gastric samples of patients with NASH as compared to those without NASH (Table 2).

### Gene expression signature associated with advanced hepatic inflammation

When samples collected from patients with advanced hepatic inflammation (score ≥ 3) were compared to that of patients with mild inflammation, 21 genes (0.001 < p < 0.05) were found to have increased gene expression (fold change range: 2.1 – 6.9) (Table 2). Among these genes, expression levels of *ADRA2B*, *CNR1* and *LEP* were also found to be correlated (r > 0.5, p < 0.05) with the degree of hepatic inflammation (Table 3). Additionally, expression levels of *IL1A* and *OPRM1* were also correlated with the degree of hepatic inflammation (r > 0.5, p < 0.05) (Table 3).

**Table 3:** Analysis of correlations between expression levels of various genes and scored characteristics of NAFLD

**Gene**	**Correlation (r)**	**p value**
**NASH**		
*IL1R1*	0.42	0.03
*OPRM1*	0.43	0.034
*SIGMAR1*	0.51	0.009
*THRB*	0.40	0.05
*ZFP91*	0.43	0.03
**Degree of inflammation**		
*ADRA2B*	0.45	0.02
*CNR1*	0.43	0.03
*LEP*	0.40	0.04
*IL1A*	0.43	0.03
*OPRM1*	0.42	0.03
**Fibrosis**		
*GHR*	0.42	0.03
*IL1A*	0.48	0.01
**Gastritis**		
*DRD1*	0.46	0.02
*GHRL*	0.41	0.04
**BMI**		
*CPE*	0.47	0.01
*ZFP91*	−0.48	0.01
**Fasting glucose (mg/dL)**		
*AGRP*	0.52	0.008
*NMB*	0.41	0.04
*THRB*	0.55	0.005
*TNF*	0.42	0.04
*UCP1*	0.65	0.0005
**Serum triglycerides (mg/dL)**		
*ADCYAP1R1*	0.48	0.01
*ADIPOQ*	0.50	0.01
*APOA4*	0.44	0.02
*CNTFR*	0.41	0.04
*CALCA*	0.44	0.02
*DRD2*	0.49	0.01
*GCGR*	0.43	0.03
*GH2*	0.41	0.04
*GLP1R*	0.56	0.003
*IL6*	0.42	0.03
*NMUR1*	0.48	0.01
*NTRK2*	0.40	0.04
*PPARGC1A*	0.42	0.04
*PRLHR*	0.43	0.03
*CPD*	−0.42	0.03

### Gene expression signature associated with hepatic fibrosis

Comparison of gastric samples collected from patients with fibrosis and samples from those without fibrosis showed the mRNAs for *NTS* and *OPRK1* genes were more than 5-fold upregulated in presence of fibrosis (p < 0.02) (Table 2). Analysis of correlations showed that mRNA levels for *GHR* and *IL1A* genes consistently increase along with a progression of fibrosis (r > 0.5, p < 0.05) (Table 3).

### Association of gene expression with risk factors for NAFLD

The levels of mRNA expression for *CPE* were correlated positively to BMI, while mRNA expression levels for *ZFP91* and BMI were correlated negatively (P < 0.01) (Table 3). Furthermore, fasting glucose levels were positively correlated with expression levels of mRNAs encoded by *AGRP*, *NMB*, *THRB*, *TNF* and *UCP1* genes Serum triglyceride levels was positively correlated with expression levels of 14 genes (Table 3), including *ADIPOQ* and *APOA4* and negatively correlated with that of *CPD* (Table 3).

## Discussion

Obesity is commonly viewed as an accumulation of excessive number of enlarged adipocytes within an abdominal cavity and in subcutaneous depots. In addition, obesity is also associated with fat accumulation in a number of organs, in particular, muscle and liver [20]. These ectopic sites are not well adapted for fat storage and even modest increases in lipid concentrations can be manifested as tissue dysfunction due to lipotoxicity [21]. A spectrum of lipotoxicity-associated changes in expression patterns has been demonstrated both in muscle and liver of obese patients [22-24] as well as in the animal models of obesity [25].

In addition to fatty infiltration of these organs, a number of other changes can happen in obese patients. For instance, the gastric tissue of obese individuals undergoes changes that are visible by histologic assessment. In fact, oxyntic mucosa of morbidly obese patients without metabolic syndrome contains more ghrelin-immunoreactive cells as compared to that of non-obese subjects [26]. Furthermore, serum levels of three protein products of ghrelin gene (acylated ghrelin, des-acylated ghrelin, and obestatin) have been shown to be elevated in obese patients with NAFLD [10,27]. Finally, in a previous study, we have shown that mRNAs encoding for various soluble molecules are overproduced in the gastric tissue of morbidly obese patients with advanced forms of NAFLD.

In this study, we assessed the gene expression patterns for energy metabolism-related genes in the gastric tissue of morbidly obese patients with NAFLD. In particular, we observed that accumulation of the fat in the liver parenchyma is accompanied by downregulation of genes encoding for carboxypeptidase E (*CPE*) and Interleukin 1B (*IL1B*) in the gastric mucosa of same patient. Given the proximity and intimate interaction of stomach with liver, we believe these observations in the gastric tissue may have important implications for changes seen in the hepatic tissue.

Carboxypeptidase E is involved in the post-translational processing of many prohormones and neuropeptides, including those expressed predominantly in gastrointestinal tract and playing a central role in energy homeostasis [28]. In models animals, inactivating of both CPE alleles result in obesity that is caused by defective nutrient partitioning rather than by increased food consumption [28]. Not much is known about an expression of CPE in humans, however, there are indications that allelic variations in this gene may contribute to coronary atherosclerosis, another complication of obesity and metabolic syndrome [29]. To date, ours is the first report to link the decrease in human non-adipose peripheral tissue expression of CPE to obesity related condition.

Another mRNA downregulated in gastric tissue of patients with high grade hepatic steatosis encodes for Interleukin 1 beta (*IL1B*), an inflammatory cytokine with anorexigenic function [30,31]. IL1β inhibits gene expression of orexigenic ghrelin [32] and suppresses the production of gastric acid and gastrin [32]. However, it was shown recently that IL1β supports ectopic fat accumulation in hepatocytes and adipose-tissue macrophages [33]. Interestingly, high-fat-fed (HFF) mice exhibited a preferential increase of IL-1β concentration in portal compared to systemic blood [34], thus, indicating that the liver may differ in its IL-1β regulation from other perihearl tissues.

It important to note that obesity is known to be associated with low grade systemic inflammation. Furthermore inflammation plays an important role in the pathogenesis of progressive NAFLD or NASH. In our study, the levels of 21 genes were significantly altered in the gastric tissue of NAFLD patients with hepatic inflammation (Table 2). Amongst these, *ADRA2B*, *CNR1*, *LEP* also significantly correlated (r = 0.5, p < 0.05) with the degree of hepatic inflammation.

Another interesting finding of our study relates to expression of leptin in the gastric tissue. The secretion of the leptin is acutely increased in response to inflammation and inflammatory cytokines such as TNF-α and IL-1β. Although, the major site of leptin production is white adipose tissue, the leptin gene expression has also been detected in the gastric epithelium and in the glands of the gastric fundic mucosa in both rats [35], and humans [36]. There is a body of evidence implicating the leptin in the pathogenesis of NAFLD. In a study by Chitturi et al., the leptin levels in patients with biopsy proven NASH were twice those found in non-NASH matched controls [37]. Our measurement of *LEP* mRNA in the gastric tissue of patients with various stages of NAFLD showed similar trends. *LEP* was upregulated in the in gastric tissue of patients with advanced hepatic inflammation and positively correlated with the inflammation scores of patients with NAFLD (Table 3). It is important to note that the leptin also functions as an anorexigenic hormone as it suppresses appetite [38]. Thus, an increase in its production within gastric mucosa may be a compensatory response to metabolic shift of obesity. Alternatively, observed increase in *LEP* expression may serve as an indicator of leptin tolerance or leptin resistance which is commonly reported in obese individuals [39].

It is also interesting to note that the leptin has also been shown to stimulate gene expression of neurotensin (*NTS*), a neuropeptide that is known for its expression in the small intestine [40] and for its involvement in food intake and glucose homeostasis in the peripheral tissues [41]. Furthermore, receptors to neurotensin are present at the membranes of hepatocytes. It is postulated that neurotensin protects stressed liver parenchyma as evident from reduced ALT levels and hepatic oxidative stress and increase in hepatocyte proliferation in NTS-treated cholestatic model animals [42]. In our study an increase in expression of *NTS* was also seen in stomach samples of subjects with liver fibrosis as compared to those with no/mild fibrosis (Table 2). It is thus, plausible that gastric neurotensin enters the liver via the portal vein and alleviates liver fibrosis. It is also possible that gastric-produced neurotensin may, at least in part, counteract pro-fibrotic action of its inducer, the leptin in the hepatic parenchyma.

Another important gene with expression of our NAFLD patients with significant inflammation is *CNR1*/*CB1*. Endocannabinoid receptor CNR1 is known for its expression throughout the gastrointestinal tract [43]. CNR1 is implicated in *de novo* fatty acid and cholesterol biosynthesis, in both the liver and the adipose tissue [44]. In mouse models, an activation of this receptor in the liver increases *de novo* synthesis of fatty acids [45]. Furthermore, CNR1 antagonists improve metabolic parameters by exerting an anorexigenic effect, suppressing lipogenesis and reducing inflammation [44]. Peripheral CNR1 blockade has also been shown to reverse leptin resistance commonly seen in obese patients. This reversal is mediated by a decrease in leptin secretion through adipose tissue [46]. Thus, the concomitant increase in expression of gastric *CNR1* and *LEP* in patients with hepatic inflammation seen in this study may be another indication of contribution of peripheral leptin resistance to obesity-associated NASH. On the other hand, CNR1 was recently implicated in the pathophysiology of acute and chronic liver conditions, including inflammation, fibrogenesis and steatosis [47]. While faintly expressed in normal livers, CNR1 undergo substantial up-regulation after liver injury induced by various causes [48]. It is tempting to speculate that observed increase in expression of gastric CNR1 in morbidly obese patients with advanced hepatic inflammation may point to a similar regulatory process within the stomach and the liver under conditions of positive energy balance.

**Figure 1:**
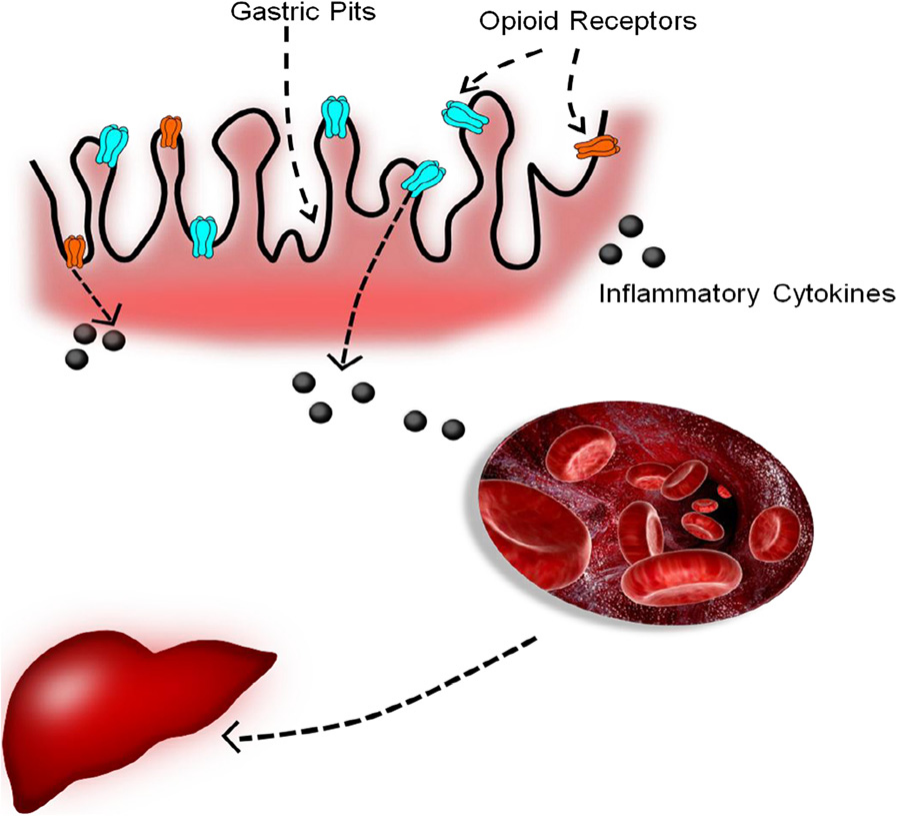
**Increased expression of opioid receptors within gastric mucosa may trigger the release of inflammatory cytokines into circulation.** The close proximity of liver to stomach and thus the secreted cytokines results in increased hepatic inflammation.

Another interesting finding of our study is related to an increase in the gene expression of opioid receptors *OPRM1* and *OPRK1* (Figure 1). Involvement of opioid receptors in the development of obesity has been widely recognized. This is potentially due to well-documented anorexigenic effects of administering general opioid receptor antagonists [49]. Interestingly, recent studies have also implicated opioid receptors in inflammation. In fact, polymorphism in the human mu-opioid receptor OPRM1 gene was associated with baseline proinflammatory cytokine levels in healthy subjects [50]. We have previously shown an altered inflammatory cytokine profiles in the gastric tissue of morbidly obese patients with NAFLD [19]. It is possible that an increase in the opioid signaling within gastric mucosa results in a shift to proinflammatory environment within this organ. In turn, an increase in the gastric production of proinflammatory cytokines may contribute to systemic inflammation and augment inflammatory processes in hepatic parenchyma (Figure 1).

## Conclusions

**Figure 2:**
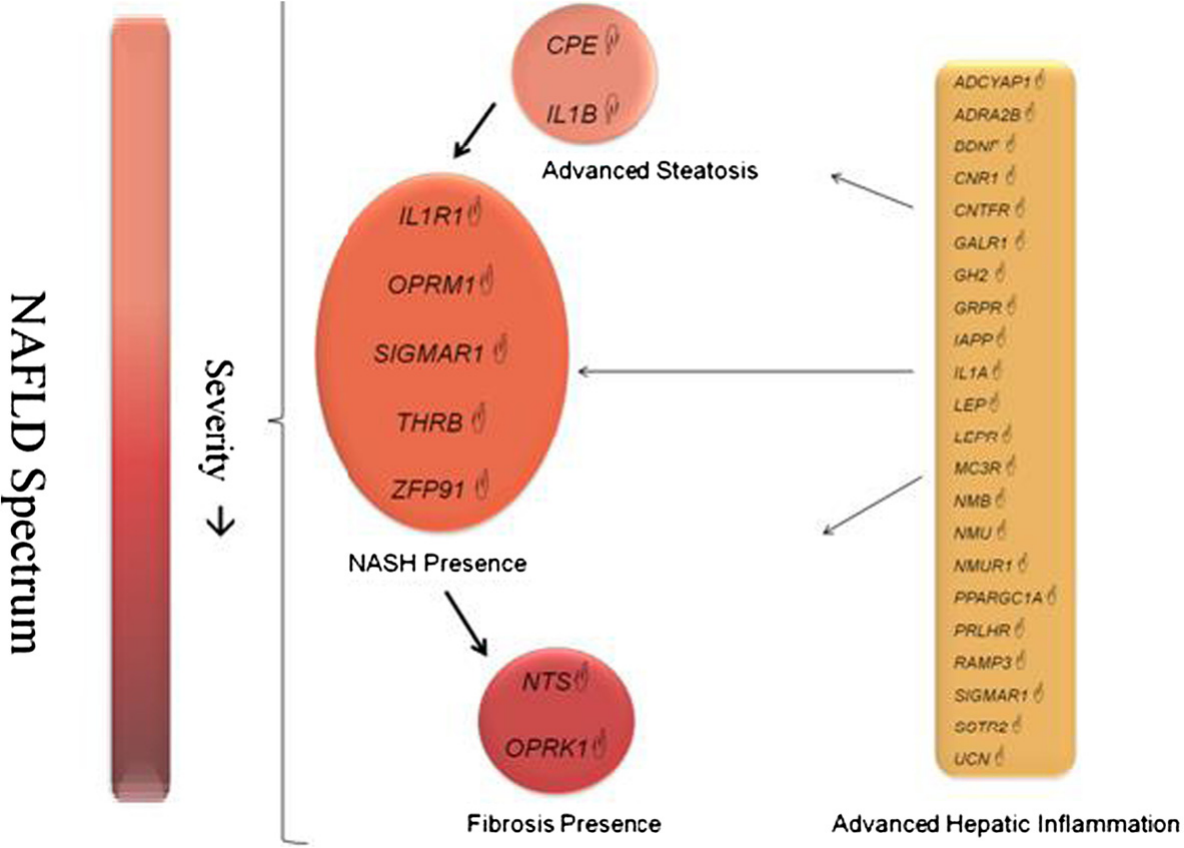
**Model depicting the changes in the expression landscape in gastric tissue of obese patients with NAFLD.** Differentially expressed genes (p < 0.05) are unique and non-overlapping among the cohorts analyzed. Genes showing fold up-regulation are indicated by ☝ symbol, while genes showing a fold down-regulation are indicated by ☟ symbol.

In conclusion, we have shown differential expression of energy metabolism associated genes in the gastric tissue of obese NAFLD patients. Importantly, these gene expression profiles are associated with changes in the hepatic parenchyma as reflected in increased scores for hepatic steatosis, inflammation, fibrosis and NASH (Figure 2). Importantly, we see that the gene expression changes in stomach are distinct and non-overlapping in different stages of NAFLD. This study suggests the complex interplay of multiple organs in the pathogenesis of obesity-related complications such as NAFLD and provides further evidence supporting an important role for gastric tissue in promoting obesity-related complications.

## Competing interests

The authors declare that they have no competing interests.

## Authors’ contributions

RM performed qPCRs and produced figures; ABir, ABar and RM wrote the manuscript; LW and RM performed statistical analysis; ZaY collected samples and performed chart reviews; AM and HE are bariatric surgeons who collected samples according to excusion and inclusion criteria; ZG reviewed and scored histological slides of the liver; ABar, ABir, VC and ZoY designed experiments and edited the manuscript. All authors read and approved the final manuscript.

## Pre-publication history

The pre-publication history for this paper can be accessed here:

http://www.biomedcentral.com/1471-230X/14/72/prepub

## Supplementary Material

Additional file 1: Table S1 Metabolism related genes profiled for their expression levels in fundic gastric samples of 24 obese subjects. Click here for file
