# Supplementary material for: Expression of energy metabolism related genes in the gastric tissue of obese individuals with non-alcoholic fatty liver disease
Source: BMC Gastroenterol. 2014 Apr 9;14:72. doi: 10.1186/1471-230X-14-72 (PMC4021272; doi:10.1186/1471-230X-14-72)
Supplement: Additional file 1: Table S1 — Metabolism related genes profiled for their expression levels in fundic gastric samples of 24 obese subjects. [file 1471-230X-14-72-S1.docx]

**Supplementary Table 1.** Metabolism related genes profiled for their expression levels in fundic gastric samples of 24 obese subjects

| **Gene Description** | **Symbol** | **Function** |
| --- | --- | --- |
| **Genes Related to Energy Expenditure** | | |
| Adenylate cyclase activating polypeptide 1 (pituitary) | *ADCYAP1* | Stimulates adenylate cyclase and subsequently increases the cAMP level in target cells. |
| Adenylate cyclase activating polypeptide 1 (pituitary) receptor type I | *ADCYAP1R1* | May regulate the release of adrenocorticotropin, luteinizing hormone, growth hormone, prolactin, epinephrine, and catecholamine. May play a role in spermatogenesis and sperm motility. Causes smooth muscle relaxation and secretion in the gastrointestinal tract |
| Adiponectin, C1Q and collagen domain containing | *ADIPOQ* | Encoded protein circulates in the plasma and is involved with metabolic and hormonal processes. Involved in the control of fat metabolism and insulin sensitivity, with direct anti-diabetic, anti-atherogenic and anti-inflammatory activities. Stimulates AMPK phosphorylation and activation in the liver and the skeletal muscle, enhancing glucose utilization and fatty-acid combustion. Antagonizes TNF-alpha by  negatively regulating its expression in various tissues such as liver and macrophages, and also by counteracting its effects. Inhibits endothelial NF-kappa-B signaling through a cAMP-dependent pathway. |
| Adiponectin receptor 1 | *ADIPOR1* | Receptor for adiponectin. Mediates increased AMPK, PPARA ligand activity, fatty acid oxidation and glucose uptake by adiponectin. |
| Adiponectin receptor 2 | *ADIPOR2* | Receptor for adiponectin. Mediates increased AMPK, PPARA ligand activity, fatty acid oxidation and glucose uptake by adiponectin. |
| Adrenergic, beta-1-, receptor | *ADRB1* | Beta-adrenergic receptor, mediates the catecholamine-induced activation of adenylate cyclase through the action of G proteins. This receptor binds epinephrine and norepinephrine. |
| Complement component 3 | *C3* | Central role in the activation of the complement system. Derived from proteolytic degradation of complement C3, C3a anaphylatoxin is a mediator of local inflammatory process. |
| Carboxypeptidase D | *CPD* | Regulatory B-type carboxypeptidase belonging to metallocarboxypeptidase family of enzymes. |
| Carboxypeptidase E | *CPE* | Involved in the biosynthesis of peptide hormones and neurotransmitters, including insulin. |
| Peroxisome proliferator-activated receptor alpha | *PPARA* | Nuclear transcription factor, key regulator of lipid metabolism. Regulates the peroxisomal beta-oxidation pathway of fatty acids. |
| Peroxisome proliferator-activated receptor gamma | *PPARG* | Nuclear transcription factor. Regulator of adipocyte differentiation and glucose homeostasis. Controls the peroxisomal beta-oxidation pathway of fatty acids. Implicated in the pathology of numerous diseases including obesity, diabetes, atherosclerosis and cancer. |
| Peroxisome proliferator-activated receptor gamma, coactivator 1 alpha | *PPARGC1A* | Interacts with PPARg. Transcriptional coactivator for steroid receptors and nuclear receptors. Regulate the activities of, cAMP response element binding protein (CREB) and nuclear respiratory factors (NRFs). Can regulate key mitochondrial genes that contribute to the program of adaptive thermogenesis. |
| Uncoupling protein 1 (mitochondrial, proton carrier) | *UCP1* | Mitochondrial uncoupling proteins, expressed only in brown adipose tissue, a specialized tissue which functions to produce heat. |
| Protein tyrosine phosphatase, non-receptor type 1 | *PTPN1* | Negative regulator of insulin signaling by dephosphorylating the phosphotryosine residues of insulin receptor kinase. dephosphorylate epidermal growth factor receptor kinase, as well as JAK2 and TYK2 kinases. |
| Sigma non-opioid intracellular receptor 1 | *SIGMAR1* | Play an important role in the cellular functions of various tissues associated with the endocrine, immune, and nervous systems. Functions in lipid transport from the endoplasmic reticulum. Also regulates ion channels like the potassium channel and could modulate neurotransmitter release |
| Thyroid hormone receptor, beta (erythroblastic leukemia viral (v-erb-a) oncogene homolog 2, avian) | *THRB* | Nuclear hormone receptor for triiodothyronine. |
| **Orexigenic Genes** | | |
| Adrenergic, alpha-2B-, receptor | *ADRA2B* | Mediate the catecholamine-induced inhibition of adenylate cyclase through the action of G proteins. |
| Agouti related protein homolog (mouse) | *AGRP* | Antagonist of the melanocortin-3 and melanocortin-4 receptor. Plays a role in weight homeostasis. |
| Cannabinoid receptor 1 (brain) | *CNR1* | Involved in cannabinoid-induced CNS effects. Acts by inhibiting adenylate cyclase. |
| Galanin prepropeptide | *GAL* | Contracts smooth muscle of the gastrointestinal and genitourinary tract, regulates growth hormone release, modulates insulin release, and may be involved in the control of adrenal secretion. |
| Galanin receptor 1 | *GALR1* | Receptor for GAL, inhibits adenylyl cyclase via a G protein of the Gi/Go family. |
| Ghrelin/obestatin prepropeptide | *GHRL* | Encodes ghrelin-obestatin preproprotein. Ghrelin has an appetite-stimulating effect, induces adiposity and stimulates gastric acid secretion. Obestatin has appetite-reducing effect resulting in decreased food intake. May reduce gastric emptying activity and jejunal motility. |
| Growth hormone secretagogue receptor | *GHSR* | Receptor for ghrelin. Stimulates growth hormone secretion. |
| Melanin-concentrating hormone receptor 1 | *MCHR1* | G protein-coupled receptor family 1. Inhibit cAMP accumulation and stimulate intracellular calcium flux. |
| Hypocretin (orexin) receptor 1 | *HCRTR1* | G-protein coupled receptor involved in the regulation of feeding behavior. |
| Neuropeptide Y | *NPY* | Implicated in the control of feeding and in secretion of gonadotrophin-release hormone |
| Neuropeptide Y receptor Y1 | *NPY1R* | Receptor for neuropeptide Y and peptide YY. |
| Nuclear receptor subfamily 3, group C, member 1 (glucocorticoid receptor) | *NR3C1* | Function both as a transcription factor and as a regulator of other transcription factors. It is involved in inflammatory responses, cellular proliferation, and differentiation in target tissues. |
| Opioid receptor, kappa 1 | *OPRK1* | Inhibits neurotransmitter release by reducing calcium ion currents and increasing potassium ion conductance. Receptor for dynorphins. May play a role in arousal and regulation of autonomic and neuroendocrine functions. |
| Opioid receptor, mu 1 | *OPRM1* | Inhibits neurotransmitter release by reducing calcium ion currents and increasing potassium ion conductance.Receptor for beta-endorphin. |
| **Anorectic Genes** | | |
| Apolipoprotein A-IV | *APOA4* | May have a role in chylomicrons and VLDL secretion and catabolism. Required for efficient activation of lipoprotein lipase. |
| Attractin | *ATRN* | Involved in the initial immune cell clustering during inflammatory response. May regulate chemotactic activity of chemokines. May play a role in melanocortin signaling pathways that regulate energy homeostasis. |
| Brain-derived neurotrophic factor | *BDNF* | May play a role in the regulation of stress response and in the biology of mood disorders. |
| Bombesin-like receptor 3 | *BRS3* | Mediates its action by association with G proteins that activate a phosphatidylinositol-calcium second messenger system. |
| Calcitonin-related polypeptide alpha | *CALCA* | Involved in calcium regulation and acts to regulate phosphorus metabolism. |
| Calcitonin Receptor | *CALCR* | Involved in maintaining calcium homeostasis and in regulating osteoclast-mediated bone resorption. |
| CART prepropeptide | *CARTPT* | Satiety factor inhibits both normal and starvation-induced feeding and completely blocks the feeding response induced by neuropeptide Y and regulated by leptin in the hypothalamus. |
| Cholecystokinin | *CCK* | Induces gall bladder contraction and the release of pancreatic enzymes in the gut. |
| Cholecystokinin A receptor | *CCKAR* | In the central and peripheral nervous system this receptor regulates satiety and the release of beta-endorphin and dopamine. |
| Colipase, pancreatic | *CLPS* | Cofactor of pancreatic lipase and has a biological activity as a satiety signal. |
| Ciliary neurotrophic factor receptor | *CNTFR* | Plays a critical role in neuronal cell survival, differentiation and gene expression. Single nucleotide polymorphisms in this gene may be associated early onset of eating disorders. |
| Corticotropin releasing hormone receptor 1 | *CRHR1* | Essential for the activation of signal transduction pathways that regulate diverse physiological processes including stress, reproduction, immune response and obesity. |
| Dopamine receptor D1 | *DRD1* | Stimulates adenylyl cyclase and activates cyclic AMP-dependent protein kinases. |
| Dopamine receptor D2 | *DRD2* | Inhibits adenylyl cyclase activity. |
| Glucagon | *GCG* | Key role in glucose metabolism and homeostasis. |
| Glucagon receptor | *GCGR* | Receptor plays a central role in regulating the level of blood glucose by controlling the rate of hepatic glucose production and insulin secretion. |
| Growth hormone 1 | *GH1* | Important role in growth control. Stimulate the liver and other tissues to secrete IGF-1. Also stimulates amino acid uptake and protein synthesis. |
| Growth hormone 2 | *GH2* | Important role in growth control. Stimulate the liver and other tissues to secrete IGF-1. Also stimulates amino acid uptake and protein synthesis. |
| Growth hormone receptor | *GHR* | On ligand binding, couples to the JAK2/STAT5 pathway. |
| Glucagon-like peptide 1 receptor | *GLP1R* | Receptor activity mediated by G proteins which activate adenylyl cyclase. Role in delaying gastric emptying and regulating appetite. |
| Gastrin-releasing peptide | *GRP* | Regulate release of gastrointestinal hormones smooth muscle cell contraction, and epithelial cell proliferation. |
| Gastrin-releasing peptide receptor | *GRPR* | Mediates its action by association with G proteins that activate a phosphatidylinositol-calcium second messenger system. |
| Hypocretin (orexin) neuropeptide precursor | *HCRT* | Encodes a hypothalamic neuropeptide precursor protein that gives rise to two mature neuropeptides, orexin A and orexin B, by proteolytic processing. Role in feeding behavior, metabolism, and homeostasis |
| Histamine receptor H1 | *HRH1* | In peripheral tissues, the H1 subclass of histamine receptors mediates the contraction of smooth muscles, increase in capillary permeability as well as mediating neurotransmission in the central nervous system. |
| 5-hydroxytryptamine (serotonin) receptor 2C | *HTR2C* | Receptor for serotonin, receptor mediates its action by association with G proteins that activate a phosphatidylinositol-calcium second messenger system. |
| Islet amyloid polypeptide | *IAPP* | Selectively inhibits insulin-stimulated glucose utilization and glycogen deposition in muscle, while not affecting adipocyte glucose metabolism. |
| Interleukin 1, alpha | *IL1A* | Produced by activated macrophages, IL-1 stimulates thymocyte proliferation by inducing IL-2 release, B-cell maturation and proliferation, and fibroblast growth factor activity. IL-1 proteins are involved in the inflammatory response. |
| Interleukin 1, beta | *IL1B* | Produced by activated macrophages, IL-1 stimulates thymocyte proliferation by inducing IL-2 release, B-cell maturation and proliferation, and fibroblast growth factor activity. IL-1 proteins are involved in the inflammatory response. |
| Interleukin 1 receptor, type I | *IL1R1* | Receptor for interleukin-1 alpha (IL-1A), beta (IL-1B), and interleukin-1 receptor antagonist protein (IL-1RA). Binding to the agonist leads to the activation of NF-kappa-B. |
| Interleukin 6 (interferon, beta 2) | *IL6* | Primarily produced at sites of acute and chronic inflammation, where it is secreted into the serum and induces a transcriptional inflammatory response through interleukin 6 receptor, alpha. |
| Interleukin 6 receptor | *IL6R* | Activation leads to the regulation of the immune response, acute-phase reactions and hematopoiesis. |
| Insulin | *INS* | Insulin decreases blood glucose concentration. It increases cell permeability to monosaccharides, amino acids and fatty acids. It accelerates glycolysis, the pentose phosphate cycle, and glycogen synthesis in liver. |
| Insulin receptor | *INSR* | Binds insulin and has a tyrosine-protein kinase activity. |
| Leptin | *LEP* | Plays a major role in the regulation of body weight. |
| Leptin receptor | *LEPR* | On ligand binding, mediates signaling through JAK2/STAT3. Involved in the regulation of fat metabolism. |
| Melanocortin 3 receptor | *MC3R* | A G-protein-coupled receptor for melanocyte-stimulating hormone and adrenocorticotropic hormone. Activation mediated by G proteins which activate adenylate cyclase. |
| Neuromedin B | *NMB* | Stimulates smooth muscle contraction |
| Neuromedin B receptor | *NMBR* | involved in the regulation of many biological functions including sensory transmission, thermoregulation, feeding, pituitary, gastric and pancreatic secretion. |
| Neuromedin U | *NMU* | Stimulates muscle contractions of specific regions of the gastrointestinal tract. |
| Neuromedin U receptor 1 | *NMUR1* | Mediate diverse biological effects including contraction of the uterus, peripheral vasoconstriction causing hypertension and activation of the HPA axis resulting in decreased food intake via the CRH system. |
| Neurotrophic tyrosine kinase, receptor, type 2 | *NTRK2* | Tyrosine-protein kinase receptor for brain-derived neurotrophic factor (BDNF), neurotrophin-3 and neurotrophin-4/5. |
| Neurotensin | *NTS* | Role in endocrine or paracrine role in the regulation of fat metabolism. |
| Neurotensin receptor 1 (high affinity) | *NTSR1* | Activates a phosphatidylinositol-calcium second messenger system. |
| Proopiomelanocortin | *POMC* | Wide range of physiological functions, including pigmentation, energy homeostasis, inflammation, immunomodulation, steroidogenesis and temperature control. |
| Prolactin releasing hormone receptor | *PRLHR* | Implicated in lactation, regulation of food intake and pain-signal processing. |
| Peptide YY | *PYY* | Gut peptide inhibits exocrine pancreatic secretion, has a vasoconstrictory action and inhibits jejuna and colonic mobility |
| Receptor (G protein-coupled) activity modifying protein 3 | *RAMP3* | Transports the calcitonin gene-related peptide type 1 receptor (CALCRL) to the plasma membrane. Acts as a receptor for adrenomedullin (AM) together with CALCRL. |
| Sortilin 1 | *SORT1* | Sorting receptor in the Golgi compartment and as a clearance receptor on the cell surface. Probably required in adipocytes for the formation of specialized storage vesicles containing the glucose transporter GLUT4. |
| Somatostatin | *SST* | Inhibits the release of somatotropin. Couples to adenylyl cyclase, PLC, K+ channels, Ca2+ channels and others. |
| Somatostatin receptor 2 | *SSTR2* | Coupled to inhibition of adenylyl cyclase. In addition it stimulates phosphotyrosine phosphatase and PLC. |
| Tumor necrosis factor | *TNF* | Secreted by macrophages. Binds to receptors TNFRSF1A/TNFR1 and TNFRSF1B/TNFBR. Involved in the regulation of wide spectrum of biological processes including cell proliferation, differentiation, apoptosis, lipid metabolism, and coagulation. |
| Thyrotropin-releasing hormone | *TRH* | Responsible for the regulation and release of thyroid-stimulating hormone, as well as prolactin. |
| Urocortin | *UCN* | Stimulates the secretion of adrenocorticotropic hormone (ACTH). |
| Zinc finger protein 91 homolog (mouse) | *ZFP91* | Belongs to zinc finger family of proteins, regulator of the non-canonical NF-kappaB pathway in lymphotoxin-beta receptor signaling. May also play an important role in cell proliferation and/or anti-apoptosis. |
| **Housekeeping Genes** | | |
| Beta-2-microglobulin | *B2M* | Used as normalization control |
| Hypoxanthine phosphoribosyltransferase 1 | *HPRT1* | Used as normalization control |
| Ribosomal protein L13a | *RPL13A* | Used as normalization control |
| Glyceraldehyde-3-phosphate dehydrogenase | *GAPDH* | Used as normalization control |
| Actin, beta | *ACTB* | Used as normalization control |
